# Reduction in Operative Time After Implementation of Computer-Assisted Navigation in Direct Anterior Total Hip Arthroplasty With Consideration of Learning Curve

**DOI:** 10.1016/j.artd.2025.101892

**Published:** 2025-11-13

**Authors:** Roy F. Small, Michelle Drouin, Mindy Flanagan, Manda Coupe, Jay Fiechter, Christopher N. Johnson

**Affiliations:** a Orthopaedics Northeast, Fort Wayne, IN; b Health Services and Informatics Research, Parkview Mirro Center for Research and Innovation, Fort Wayne, IN

**Keywords:** Computer-assisted navigation, Surgical efficiency, Operative time, Total hip arthroplasty, Learning curve

## Abstract

**Background:**

Computer-assisted navigation (CAN) in total hip arthroplasty (THA) is associated with improved component positioning accuracy. However, its impact on operative efficiency remains uncertain and may be influenced by the learning curve during adoption. This study evaluates the effect of a computerized hip navigation system on operative time and surgical complications.

**Methods:**

We conducted a retrospective chart review of 300 consecutive direct anterior THAs by a single surgeon: 150 using conventional overlay, followed by 150 after implementation of CAN. After applying exclusion criteria, 292 cases remained. Demographic and perioperative variables were collected. Generalized linear modeling adjusted for covariates (body mass index, age, American Society of Anesthesiologists class) to assess relationship between surgical technique and operative time. Postoperative complications were compared between groups.

**Results:**

CAN was associated with significant reduction in operative time. Compared to conventional THA (covariate-adjusted mean: 47.63 minutes), operative time was 2.65 minutes shorter during the learning phase, identified via statistical analysis as the first 100 CAN cases. Post-learning phase, mean operative time further decreased to 40.42 minutes, 7.21 minutes less than conventional THA (*P* < .001). Body mass index was positively associated with increased operative time; age showed a modest inverse relationship. No significant difference in complication rates was observed between conventional and CAN groups (*P* = .54).

**Conclusions:**

Implementation of CAN was associated with significantly decreased operative time after a brief learning curve, without an increase in complications. Findings support safe adoption of CAN in direct anterior THA and suggest enhanced operative efficiency, aligning with the demands of value-based care and increasing surgical volumes.

## Introduction

According to Medicare data, over 250,000 total hip arthroplasty (THA) procedures were performed in 2019 alone [1]. Projections indicate that this figure is anticipated to surpass 700,000 by the year 2040. Despite the increasing demand for total joint arthroplasty, the work relative value units assigned by the Centers for Medicare and Medicaid Services have consistently decreased over the last 2 decades [2]. Amid declining reimbursements, the implementation of bundled payment models, and the emphasis of value-based care incentives, the imperative for operative efficiency has become paramount. Intraoperative efficiency directly impacts patient care outcomes as well. Prolonged operative time has been correlated with an elevated risk of postoperative complications in arthroplasty [3,4], notably infection [5]. In an effort to enhance patient outcomes, a growing body of research has been dedicated to exploring the integration of technology in joint arthroplasty. The utilization of robotics and computer navigation has demonstrated improved accuracy and reduced outliers in acetabular component placement [6]. However, multiple studies have indicated that the adoption of such technology decreases efficiency, consequently prolonging operative time [[6], [7], [8], [9], [10]]. This has led to the development of intraoperative technologies that may be able to improve implant positioning while maintaining operative time neutrality or even improve operative efficiency.

The primary aim of this study was to evaluate the impact of the Velys Hip Navigation system (Depuy Synthes, West Chester, PA, USA) on operative time. Our primary hypothesis was that incorporating this technology would result in reduced operative duration, particularly after the learning phase for the technology had been completed. In addressing this hypothesis, we additionally sought to assess what other factors may contribute to the relationship between surgical technique and operative time, such as patient age, body mass index (BMI), or American Society of Anesthesiologists (ASA) physical status score. Secondarily, we also assessed the relationship between surgical technique and postoperative complications. Through this analysis, we sought to support surgeons in adopting advanced technologies, contribute to the expanding body of research on technology in total joint arthroplasty, and showcase how integrating such innovations can optimize efficiency in THA procedures.

## Material and methods

A retrospective chart review was conducted on 300 consecutive patients who underwent direct anterior THA (DA THA) performed by the senior author. This cohort consisted of the 150 patients treated immediately before the implementation of the Velys Hip Navigation system at our institution and the first 150 patients treated after its adoption. The senior author had previously performed over 250 DA THA procedures, exceeding the estimated learning curve for this approach [[11], [12], [13], [14]]. Patients were included if they were over 18 years of age and underwent DA THA. Exclusion criteria included patients under 18 years old, conversion hip arthroplasty, cemented femoral component, Crowe 3 or 4 hip dysplasia, THA utilizing an approach other than DA, and revision THA. Of 300 surgeries, 8 were excluded from analyses for the following reasons: age less than 18 years old (n = 1), cemented prostheses (n = 3), and Crowe 3 hip dysplasia (n = 4). Patients included in the sample had an average age of 63.0 years (SD = 9.8, range = 32.0, 88.0) and average BMI of 30.8 kg/m^2^ (SD = 5.7, range = 18.0, 42.7). Nearly all patients (91.1%, 266/292) had preoperative diagnosis of primary osteoarthritis. Sample characteristics are displayed in Table 1.

**Table 1:** Sample characteristics.

Characteristic	Surgical group		Total (N = 292)	*P* value
	Conventional THA (N = 145)	CAN THA (N = 147)		
Age (y)				
Mean (SD)	62.21 (9.39)	63.80 (10.08)	63.01 (9.76)	.16
Sex, n(%)				
Female	057 (39.3%)	086 (58.5%)	143 (49.0%)	.001^b^
Male	088 (60.7%)	061 (41.5%)	149 (51.0%)	
BMI				
Mean (SD)	31.11 (6.07)	30.48 (5.21)	30.79 (5.65)	.34
Side, n(%)				
Left	059 (40.7%)	065 (44.2%)	124 (42.5%)	.54
Right	086 (59.3%)	082 (55.8%)	168 (57.5%)	
ASA PS score^a^, n(%)				
2	100 (69.0%)	106 (72.1%)	206 (70.5%)	.65
3	044 (30.3%)	041 (27.9%)	085 (29.1%)	
4	001 (00.7%)	000 (00.0%)	001 (00.3%)	
Preoperative diagnosis^a^, n(%)				
Secondary OA/Legg-Calve-Perthes	001 (00.7%)	001 (00.7%)	002 (00.7%)	.002^b^
Femur avascular necrosis	016 (11.0%)	003 (02.0%)	019 (06.5%)	
Femur avascular necrosis and troch bursitis	001 (00.7%)	000 (00.0%)	001 (00.3%)	
Fracture with nonunion	001 (00.7%)	000 (00.0%)	001 (00.3%)	
Hip fracture	001 (00.7%)	000 (00.0%)	001 (00.3%)	
Pelvis avascular necrosis	000 (00.0%)	001 (00.7%)	001 (00.3%)	
Primary OA	125 (86.2%)	141 (95.9%)	266 (91.1%)	
Primary OA and metastatic prostate cancer	000 (00.0%)	001 (00.7%)	001 (00.3%)	

OA, osteoarthritis; PS, physical status.

a sparse table, Fisher exact test for *P* value.

b *P* value less than 0.01.

All patients in the cohort underwent DA THA using a standard anterior approach with the Hana table (Mizuho OSI, Union City, CA, USA). Preoperative templating was performed for all cases. Prior to the implementation of the Velys Hip Navigation system, a conventional overlay method was employed to determine leg length and offset intraoperatively. Velys is an intraoperative image-based navigation tool that utilizes fluoroscopic radiographs and user-defined reference points to compare preoperative and intraoperative radiographs. It provides real-time data on acetabular inclination, acetabular version, leg length, and offset. Operative time, defined from skin incision to wound closure, included all registration steps. Because the Velys system does not require intraincisional registration, pins, or arrays, the workflow is consistent across body habitus, and any time savings would reflect intraoperative efficiencies compared to the conventional overlay method. Preoperative templating was performed utilizing similar software in both groups, but this was not included in operative time.

Baseline demographic data were collected for the 150 consecutive patients treated before and after the implementation of Velys, including age, sex assigned at birth, and BMI. Surgical data were also gathered, including preoperative diagnosis, procedure details, use of intraoperative computer-assisted navigation (CAN), operative time, and postoperative complications or readmissions. The date range for the index procedures in this cohort was February 19, 2020, to March 3, 2022. The Velys system was implemented on April 21, 2021, and all DA THA procedures performed on or after this date utilized the system. For the purpose of these analyses (ie, complication and readmission rate), the duration of follow-up for each patient was 90 days after surgical date. All study procedures were reviewed by the author’s institutional review board and received an exempt determination.

### Data analyses

Surgeries were classified into the following 3 groups: 1) conventional THA; 2) learning phase CAN THA; and 3) CAN THA. Consistent with findings about learning curves for THA, learning phase CAN was operationalized as the first 100 surgeries conducted using CAN for THA (April 21, 2021–November 18, 2021) [[11], [12], [13], [14]].

Descriptive statistics (means, standard deviations for continuous variables; frequencies and percentages) were calculated for the entire sample and by group. A scatter plot of operative time by date was created to explore the overall form of the relationship between these variables. Levene’s test of homogeneity of variance was conducted to detect a difference in variability for operative time across the 3 study groups [15]. We employed generalized linear models (using a normal distribution) to test the impact of CAN on THA operative time. Tests for violation of model assumptions were conducted (normality, heteroscedasticity). Age, BMI, sex, ASA score, and preoperative diagnosis were tested as covariates and only those significant (*P* < .05) were retained and combined with the surgical group variable in the final model. Adjusted means, incorporating covariates, were calculated for operative time by surgical group and by obesity category (BMI <30, 30-34.9, 35-39.9, ≥40; <35, ≥35). Pairwise comparisons among surgical groups and obesity categories in terms of operative time were conducted. Complications related to surgical procedure were extracted, and the overall rate of complications was compared between the conventional THA and CAN THA (including learning phase) using chi-square test of independence. For these analyses, no intraoperative or postoperative component positioning evaluation or comparison was completed. The data analysis for this study was generated using SAS 9.4 software. Copyright © 2016 SAS Institute Inc. SAS and all other SAS Institute Inc. product or service names are registered trademarks or trademarks of SAS Institute Inc., Cary, NC, USA.

## Results

**Figure 1:**
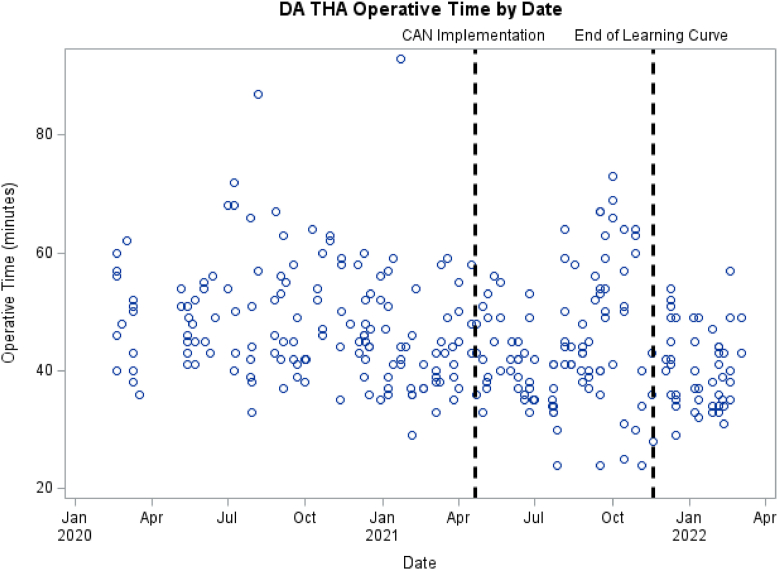
Operative time (minutes) by surgery date for DA THA (direct anterior approach total hip arthroplasty).

On average, 12.5 (SD = 4.1; range: 6 to 19) DA THA surgeries were completed per month and ranged from 1 to 7 surgeries conducted per day. Figure 1 displays operative time by date and suggests shorter operative times for the segment after the CAN learning phase ended compared to the other segments of time, which was confirmed in the results of our linear models described below. The test for homogeneity of variance was not statistically significant (*P* = .09), but, with inspection of standard deviations, suggested a trend toward reduced variability in operative time with CAN THA (SD = 6.8) vs conventional THA (SD = 9.9) and learning phase of CAN THA (SD = 10.8).

**Figure 2:**
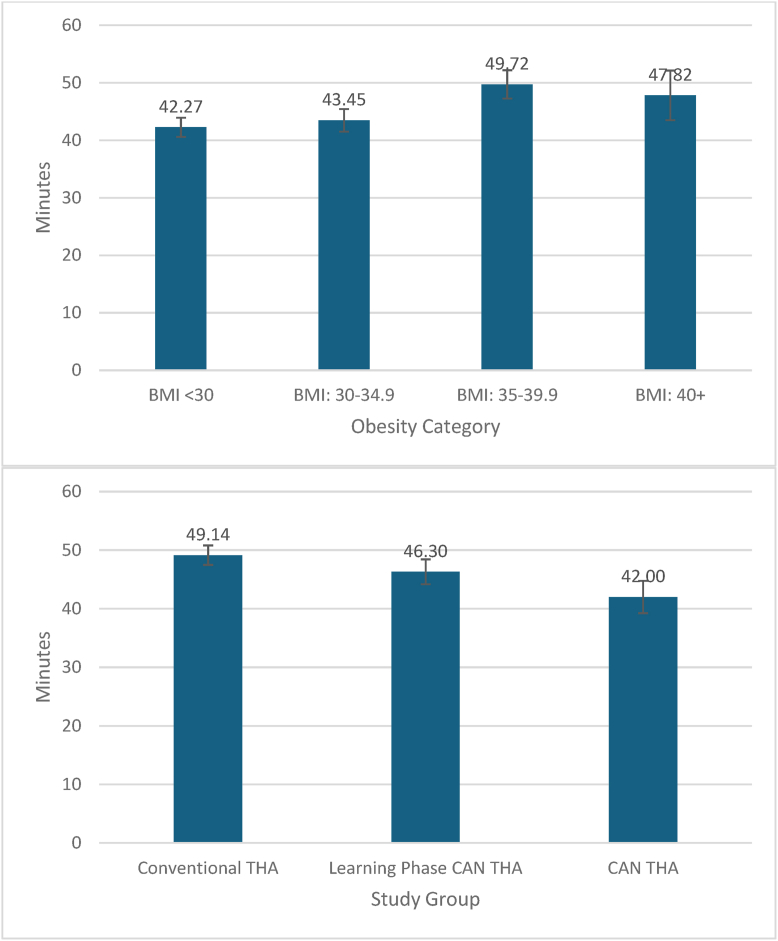
Covariate-adjusted mean operative time (minutes) and 95% confidence interval by obesity category (top panel) and study group (bottom panel).

BMI, age, and study group were significantly related to THA operative time (see Table 2). Age was inversely related to operative time, suggesting that as age increased operative time decreased. In contrast, BMI was positively related to operative time, such that with increased BMI operative time correspondingly increased in time (see Fig. 2). In pairwise comparisons, obesity categories with BMI equal to or greater than 35 had significantly longer operative times than normal or overweight categories (BMI <35; see Supplemental Table 1), with the exception that the difference between the overweight group (BMI: 30-34.9) and morbidly obese group (BMI: 40+) approached significance, *P* = .07. In contrast, operative time did not differ between the normal weight and overweight groups or the obese and morbidly obese groups.

**Table 2:** Summary results from generalized linear effects model predicting DA THA operative time (minutes).

Fixed effect	Estimate	Standard error	*Chi*-square	*P* value
Age	−0.18	0.05	10.03	.002
BMI	0.54	0.09	30.62	<.0001
Group: conventional THA vs CAN THA	7.21	1.53	22.07	<.0001
Learning phase CAN THA vs CAN THA	4.56	1.61

CAN THA operative times were 4.56 minutes less than learning phase CAN THA operative times (*P* = .005) and 7.21 minutes less than conventional THA operative times (*P* < .0001), both statistically significant differences (see Supplemental Table 2 and Fig. 2). Also significantly different, operative times for learning phase CAN THA were 2.65 minutes less than operative times for conventional THA surgeries (*P* = .03).

Complications related to surgical procedure are shown in Table 3. No differences in rates of complications were found between conventional THA and CAN/learning phase CAN THA, *Χ*^2^(1) = 0.38, *P* = .54.

**Table 3:** Complications count by conventional and CAN THA.

Complication	Conventional THA (n = 145)	CAN THA (n = 147)
Dislocation	2 (1.4%)	0 (0.0%)
Incision complication	4 (2.8%)	1 (0.7%)
Postoperative cardiac arrest	0 (0.0%)	1 (0.7%)
Pulmonary embolism	1 (0.7%)	0 (0.0%)
Acute deep infection	0 (0.0%)	3 (2.0%)
Total	7 (4.8%)	5 (3.4%)

## Discussion

This study demonstrates that the implementation of Velys Hip Navigation system was associated with a significant reduction in operative time for DA THA without an increase in complication rates. After the learning phase, mean estimated operative time decreased by over 7 minutes compared to conventional overlay technique. These findings support that CAN can be safely adopted without compromising operative time.

Our analysis supports the presence of a brief learning curve associated with adopting the Velys Hip Navigation system. The initial 100 cases utilizing Velys (the “learning phase”) demonstrated intermediate operative times between the conventional overlay technique and our learned CAN group. Notably, even during the learning phase, operative times were statically shorter than compared to the conventional overlay technique, suggesting a relatively rapid integration of the system into routine workflow. Importantly, because this technology is operated in part by the company representative, the learning curve also reflects workflow adaptation by this representative, as both the surgical team and industry partner gain experience in seamlessly integrating the system into the operative environment.

As part of our secondary analysis, patient-specific factors were also independently associated with surgical duration. BMI was positively correlated with increased operative time, consistent with prior literature that demonstrates higher technical difficulty in obese patients undergoing THA [[16], [17], [18]]. Interestingly, Velys may offer unique advantages in this cohort by providing real-time, imaging-based metrics even when intraoperative landmarks are obscured. Age also demonstrated a modest inverse relation to operative time. While the etiology of this finding is less clear, it may reflect increased muscle mass in the younger population, technically complicating the exposure during DA approach. It is worth noting that there was a statically significant difference in the sex distribution between the conventional CAN THA groups, with a higher proportion of female patients in the CAN cohort. In a sensitivity analysis with gender retained in the model, sex did not influence operative time and the same significant pairwise differences between groups were observed with equivalent estimated operative times.

Prolonged operative time has been well-documented as a risk factor for postoperative complications including infection, venous thromboembolism, and increased blood loss [5,[19], [20], [21]]. Importantly, in addition to the lack of increase in operative time, complication rates in this study were overall low and were not significantly different between patients who underwent THA with and without the use of CAN. This finding reinforces the safety profile of this technology. While we did notice 3 deep infections in the CAN group, the small number of cases suggests this is an unrelated and incidental finding. We additionally had no incidence of dislocation in the CAN group. Overall, the data support that implementing CAN in DA THA can be done safely, without elevating risk to patients. When introducing new technologies, there is always a theoretical risk of infection if additional trays or instruments are required; however, the Velys system is purely imaging-based with analysis performed away from the operative field, which minimizes this concern. Surgeons should nonetheless remain vigilant when adopting new technologies to ensure that workflow changes do not inadvertently impact sterility.

Overall, our findings suggest that the integration of such navigation systems does not necessitate longer operative times, which are frequently cited as a drawback of the use of intraoperative technologies. Considering the projected rise in arthroplasty case volume alongside declining reimbursement, even modest reduction in operative time can have a meaningful impact on operating room efficiency, resource utilization, and bundled payment models. Though robotic systems and CAN have upfront costs, reductions in operative time may offset these expenses. More importantly, in terms of patient care, these systems have consistently been shown to improve accuracy in the operating room. Specifically, CAN has been shown to provide accurate, real-time feedback on component positioning, leg length, and offset without the need for preoperative imaging [22,23]. Though assessment of financial metrics and positioning accuracy were not included in this analysis in conjunction with operative time, this represents an important area of future investigation with regard to this and other operative technologies. Although a 7-minute reduction may not directly relate to complication rates, the cumulative effect across high-volume centers can still contribute to meaningful gains in center efficiency and resource utilization.

### Limitations

Limited generalizability of the data and models should be noted. First, the data set represents a relatively small sample of patients and reflects the experience of a single surgeon and institution, so generalizing to other samples might not be appropriate, such as a sample that does not have primary osteoarthritis as the majority preoperative diagnosis. While we assessed complications over a period of 90 days, it is possible that longer-term follow-up may introduce additional complications; future research should assess this. Additionally, the retrospective nature of the analysis introduces the possibility of unmeasured confounding. Although we adjusted for age, BMI, sex, ASA score, and diagnosis, other surgical or institution variables could influence operative times. As well, the conventional THA time period spanned the COVID-19 pandemic which might have exerted some effects on operative time. Due to the sample size, the statistical approach was limited. Future studies with larger sample sizes might use interrupted time series analyses [24]. Finally, we did not assess radiographic accuracy or functional outcomes in this study.

## Conclusions

In conclusion, the use of Velys Hip Navigation in DA THA significantly reduced operative time after a brief learning curve and did not increase complication rates. In contrast to some extant literature which suggests that implementation of such technologies can extend operative time, our findings suggest that the integration of intraoperative technologies can improve efficiency while preserving safety, supporting their adoption in the context of modern value-based care.

## CRediT authorship contribution statement

**Roy F. Small:** Writing – review & editing, Writing – original draft, Project administration, Methodology, Investigation, Conceptualization. **Michelle Drouin:** Writing – review & editing, Writing – original draft, Project administration, Methodology, Conceptualization. **Mindy Flanagan:** Writing – review & editing, Writing – original draft, Visualization, Resources, Methodology, Formal analysis, Conceptualization. **Manda Coupe:** Writing – review & editing, Writing – original draft, Visualization, Project administration, Conceptualization. **Jay Fiechter:** Writing – original draft, Conceptualization. **Christopher N. Johnson:** Writing – review & editing, Supervision, Resources, Methodology, Investigation, Conceptualization.

## Conflicts of interest

C.N. Johnson reports design work royalties and consulting fees from Link Medical Products and consulting fees from J&J MedTech; all other authors declare no potential conflicts of interest.

For full disclosure statements refer to https://doi.org/10.1016/j.artd.2025.101892.

## Figures and Tables

**Supplemental Table 1:** Covariate-adjusted mean operative time (minutes) and 95% confidence interval by obesity category

Group	Estimate	95% CI
BMI <30^a^	42.27	(40.60, 43.94)
BMI: 30-34.9^a^	43.45	(41.47, 45.43)
BMI: 35-39.9^b^	49.72	(47.26, 52.19)
BMI: 40 + ^b^	47.82	(43.50, 52.14)

Estimated operative time was adjusted for age and surgical group, groups with different superscripts indicate a significant difference between groups *P* < .05. The difference between groups BMI: 30-34.9 and BMI: 40+ was marginally significant, *P* = .07.

**Supplemental Table 2:** Covariate-adjusted mean operative time (minutes) and 95% confidence interval by study group

Group	Estimate	95% CI
Conventional THA^a^	47.63	(46.15, 49.11)
Learning phase CAN THA^b^	44.98	(43.20, 46.77)
CAN THA^c^	40.42	(37.82, 43.02)

Estimated operative time was adjusted for age and BMI; groups with different superscripts indicate a significant difference between groups *P* < .05.

**Supplemental Figure 1:**
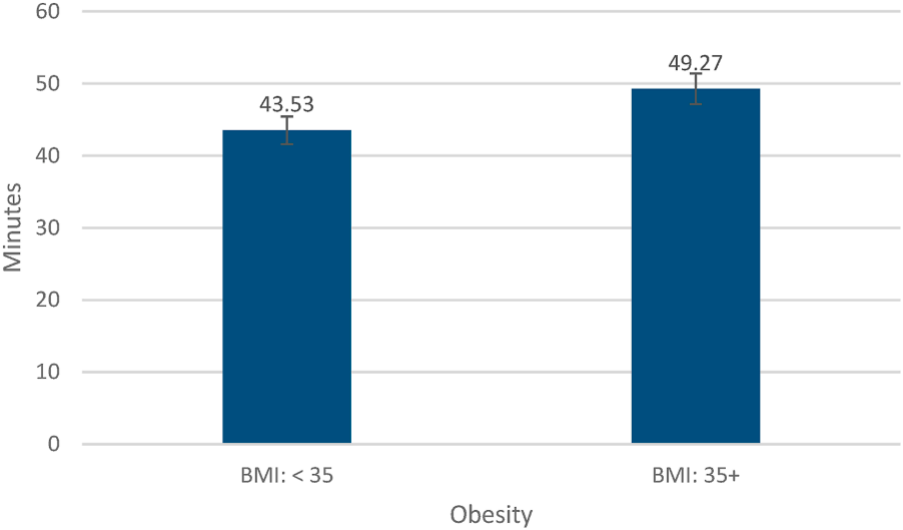
Covariate-adjusted mean operative time (minutes) and 95% confidence interval by obesity category.

## Appendix A Supplementary data

Conflict of Interest Statement for Johnson

Conflict of Interest Statement for Fiechter

Conflict of Interest Statement for Coupe

Conflict of Interest Statement for Drouin

Conflict of Interest Statement for Flanagan

Conflict of Interest Statement for Small
