# Supplementary material for: Reduction in Operative Time After Implementation of Computer-Assisted Navigation in Direct Anterior Total Hip Arthroplasty With Consideration of Learning Curve
Source: Arthroplast Today. 2025 Nov 13;39:101892. doi: 10.1016/j.artd.2025.101892 (PMC13321383; doi:10.1016/j.artd.2025.101892)
Supplement: Conflict of Interest Statement for Johnson [file mmc1.pdf]

CONFLICT OF INTEREST STATEMENT

American Association of Hip and Knee Surgeons

(Adopted from the American Academy of Orthopaedic Surgeons disclosure statement)

The following form **must be filled out completely and submitted by each author (example, 6 authors, 6 forms). All items require a response. If there is no relevant disclosure for a given item, enter "*None*."**

Reduction in Operative Time After Implementation of Computer-Assisted Navigation in Direct Anterior Total Hip Arthroplasty with Consideration of Learning Curve

Manuscript Title

1. Royalties from a company or supplier (The following conflicts were disclosed)

   Link

2. Speakers bureau/paid presentations for a company or supplier (The following conflicts were disclosed)

   None

3A. Paid employee for a company or supplier (The following conflicts were disclosed)

   None

3B. Paid consultant for a company or supplier (The following conflicts were disclosed)

   Johnson & Johnson MedTech, Link

3C. Unpaid consultants for a company or supplier (The following conflicts were disclosed)

   None

4. Stock or stock options in a company or supplier (The following conflicts were disclosed)

   NOne

5. Research support from a company or supplier as a Principal Investigator (The following conflicts were disclosed)

   None

6. Other financial or material support from a company or supplier (The following conflicts were disclosed)

   None

7. Royalties, financial or material support from publishers (The following conflicts were disclosed)

   None

8. Medical/Orthopaedic publications editorial/governing board (The following conflicts were disclosed)

   None

9. Board member/committee appointments for a society (The following conflicts were disclosed)

   None

Each author must sign AND print or type his/her name, date and submit a separate form

In addition, one BLINDED Conflict of Interest form (no author names used) should be submitted per manuscript with all author disclosures.

Christopher N Johnson	Christopher N Johnson<br>Christopher N Johnson (Aug 5, 2025 21:38:13 EDT)	Aug 5, 2025
Author Name (Print or Type)	Author Signature	Date
